# Supplementary material for: Comparative analysis of squamate brains unveils multi-level variation in cerebellar architecture associated with locomotor specialization
Source: Nat Commun. 2019 Dec 5;10:5560. doi: 10.1038/s41467-019-13405-w (PMC6895188; doi:10.1038/s41467-019-13405-w)
Supplement: Supplementary file 1 — Supplementary Information [file 41467_2019_13405_MOESM1_ESM.pdf]

Supplementary Information

Comparative analysis of squamate brains unveils multi-level variation in cerebellar architecture associated with locomotor specialization

Macrì et al.

Contents:

Supplementary Figures 1, 2
Supplementary Tables 1-8
Supplementary References

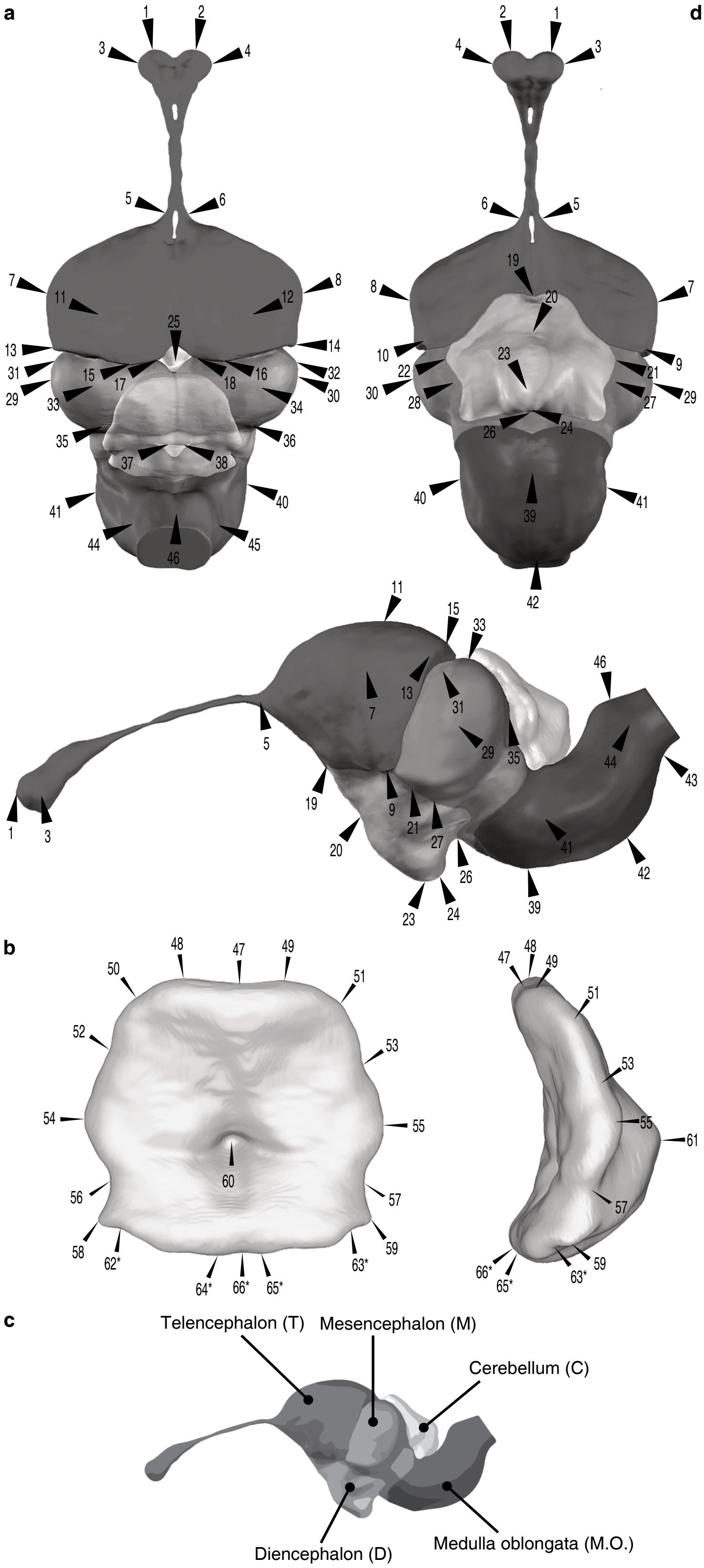

d

Landmark number	Definition	Brain region
1-2	Anterior-most extent of the olfactory bulb	T
3-4	Lateral-most extent of the olfactory bulb	T
5-6	Anterolateral margin of the cerebral hemisphere	T
7-8	Lateral-most extent of the cerebral hemisphere	T
9-10	Ventral-most extent of the cerebral hemisphere	T
11-12	Dorsal-most extent of cerebral hemisphere	T
13-14	Posterolateral-most extent of the cerebral hemisphere	T
15-16	Posterodorsal-most extent of cerebral hemisphere	T
17-18	Maximal curvature point along the posterodorsal edge of cerebral hemisphere between landmarks 15-16 and the mid-sagittal plane	T
19	Anterior-most extent of diencephalon	D
20	Optic chiasm, mid-sagittal plane	D
21-22	Lateral-most extent of the diencephalon	D
23	Ventral-most extent of diencephalon, mid-sagittal plane	D
24	Infundibulum, mid-sagittal plane	D
25	Habenular commissure, mid-sagittal plane	D
26	Mesencephalic flexure, mid-sagittal plane	D, M, M.O.
27-28	Ventral-most extent of tectum mesencephali	M
29-30	Lateral-most extent of tectum mesencephali	M
31-32	Anterolateral-most extent of tectum mesencephali	M
33-34	Dorsal-most extent of tectum mesencephali	M
35-36	Posterior-most extent of tectum mesencephali	M
37-38	Posterior-most extent of torus semicircularis	M
39	Ventral-most extent of medulla oblongata	M.O.
40-41	Lateral-most extent of medulla oblongata	M.O.
42	Maximal curvature point along the mid-sagittal plane of the ventral medulla oblongata, between landmarks 39 and 43	M.O.
43	Ventral-most extent of medulla oblongata in correspondence of foramen magnum	M.O.
44-45	Lateral-most extent of medulla oblongata in correspondence of foramen magnum	M.O.
46	Dorsal-most extent of medulla oblongata in correspondence of foramen magnum	M.O.
47	Dorsal-most (lizard) or posterior-most (snake) extent of cerebellum, homologous points as derived from embryonic medial upper rhombic lip, mid sagittal plane	C
48-49	Maximal curvature point along the cerebellar edge lateral to landmark 47	C
50-51	Maximal curvature point along the cerebellar edge below landmarks 48-49	C
52-53	Inflection point along the cerebellar edge below landmarks 50-51	C
54-55	Maximal curvature point along the cerebellar edge below landmarks 52-53	C
56-57	Dorsal-most extent of the lateral cerebellar peduncle	C
58-59	Lateral-most extent of the lateral cerebellar peduncle	C
60	Maximal convexity/concavity point on the cerebellar pial surface, mid-sagittal plane	C
61	Maximal convexity/concavity point on the cerebellar ventricular surface, mid-sagittal plane	C
62*-63*	Ventral-most extent of the lateral cerebellar peduncle	C
64*-65*	Ventral-most extent of the medial cerebellar peduncle	C
66*	Ventral-most extent of the cerebellar pial surface, mid-sagittal plane	C

*Present only on isolated cerebellum reconstructions

Supplementary Fig. 1: Definition of 3D anatomical landmarks. a, Position of 3D anatomical landmarks on the whole brain of the lizard *Agama agama* in dorsal (top left panel), ventral (top right), and lateral (bottom) views. **b**, Position of 3D anatomical landmarks on the isolated cerebellum of *Agama agama* in pial surface (left panel) and lateral (right) views. **c**, Schematic representation of the whole-brain of *Agama agama* highlighting the five major regions used for landmarking. **d**, Table showing the definition of 3D anatomical landmarks with associated number (see **a**, **b**) and brain region (see **c**).

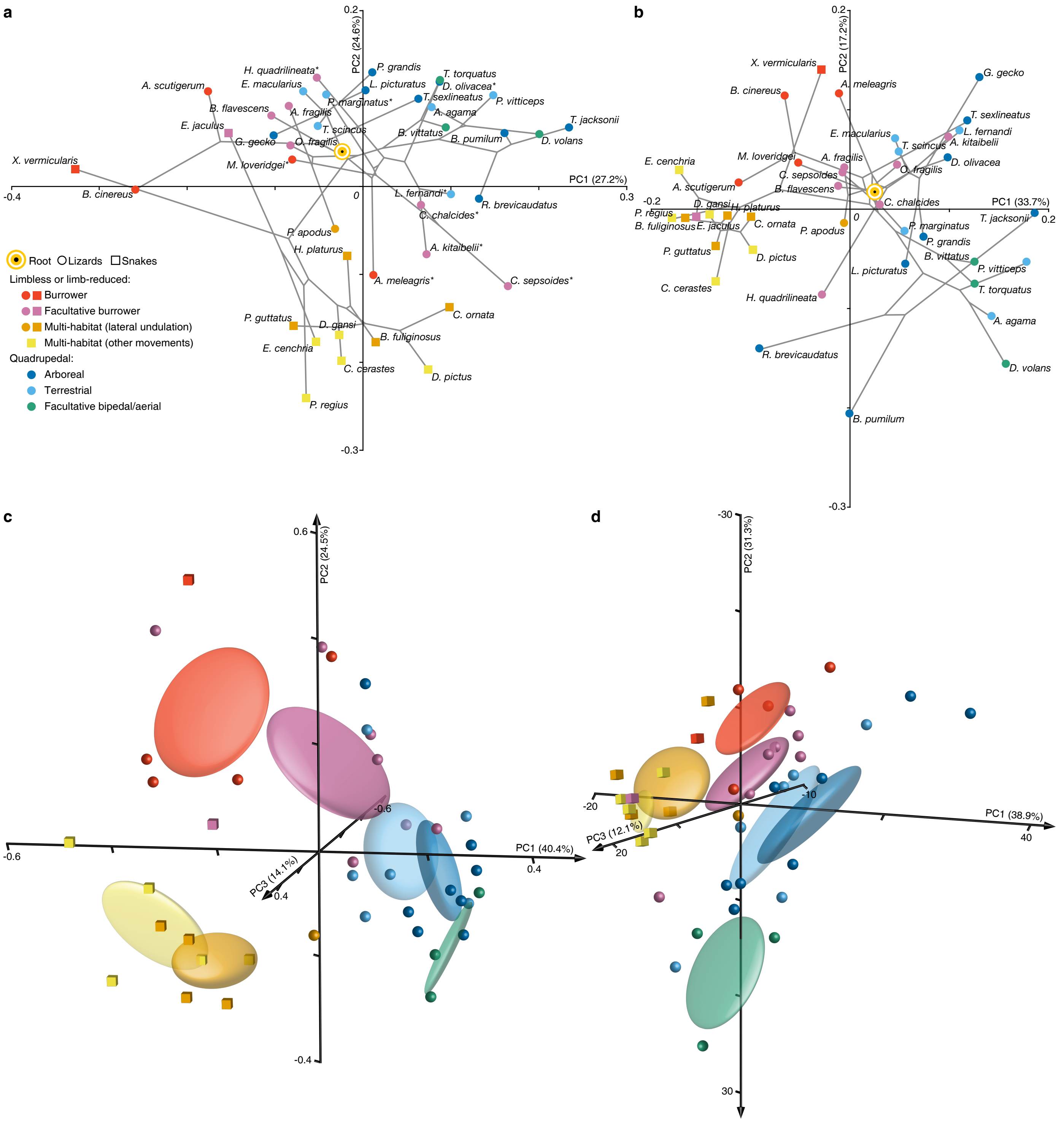

Supplementary Fig. 2: Phylomorphospace and landmark-free geometric morphometric methods. a, **b,** Plots of 2D phylomorphospace (with species names indicated) showing the cerebellar (**a**) and whole-brain (**b**) shape distribution of snakes (coloured squares) and lizards (coloured circles) with different locomotor modes (see colour code in **a**). Numbers in brackets indicate the percentage of variance explained by each of the PC axes, and 68% confidence ellipses are shown for each locomotor mode. Ancestral character estimation for each internal node was performed using squared-change parsimony, based on the most inclusive phylogenetic study available for extant squamates (see Methods), and branch lengths were derived from estimated divergence times. Asterisks in (**a**) mark individuals belonging to the *Scincidae* family. **c**, **d**, Plots of 3D morphospace obtained using landmark-free ShapeWorks (**c**) and GPSA (**d**) methods, showing the cerebellar (**c**) and whole-brain (**d**) shape distribution of snakes (coloured cubes) and lizards (coloured spheres) with different locomotor modes (see colour code in **a**). Numbers in brackets indicate the percentage of variance explained by each of the PC axes. The PC2 axis in (**d**) was inverted for better comparison with landmark-based geometric morphometric methods.

Supplementary Table 1: List of anatomical criteria, habitat modes, and movement types used to define the main locomotor categories for all squamate species (see Methods for descriptions of categorization)

Groups	Species	Families	Anatomical features	Habitat modes	Movement types	Locomotor modes	References	Specimen source*	Specimen number†	Specimen size‡
Lizard	*Ablepharus kitaibelii*	Scincidae	Slightly reduced forelimbs, elongated body	Facultative burrower	Slow lateral undulation	Limbless or limb-reduced facultative burrower	1-4	MS	3 (3D)	8.9-9.3
Lizard	*Acontias meleagris*	Scincidae	Limbless, elongated body, cylindrical skull	Burrower	Modified concertina or rectilinear	Limbless burrower	5-7	MS	3 (3D)	8.9-13.6
Lizard	*Agama agama*	Agamidae	Intact limbs	Terrestrial	Quadrupedal terrestrial	Quadrupedal terrestrial	5	FS	1 (3D)	19.7
Lizard	*Amphisbaena scutigerum*	Amphisbaenidae	Limbless, elongated body, cylindrical skull	Burrower	Modified concertina or rectilinear	Limbless burrower	3,5,6,8,9	MS, FS	3 (3D)	10.4-11.4
Lizard	*Anguis fragilis*	Anguidae	Limbless, elongated body	Facultative burrower	Slow lateral undulation	Limbless or limb-reduced facultative burrower	2-5,10	MS	2 (3D)	16.3-17.2
Lizard	*Anolis carolinensis*	Dactyloidae	Intact limbs	Arboreal	Quadrupedal arboreal	Quadrupedal arboreal	5	FS	2 (IHC)	16.1-21.0
Lizard	*Bachia flavescens*	Gymnophthalmidae	Strongly reduced limbs, elongated body	Facultative burrower	Slow lateral undulation	Limbless or limb-reduced facultative burrower	2-4,11,12	MS	1 (3D)	12.6
Lizard	*Basiliscus vittatus*	Corytophanidae	Intact limbs	Aerial/Semi-arboreal	Quadrupedal arboreal with facultative bipedal	Quadrupedal facultative bipedal/aerial	5,13	FS	3 (3D;IHC;TR)	23.9-35.8
Lizard	*Blanus cinereus*	Blanidae	Limbless, elongated body, cylindrical skull	Burrower	Modified concertina or rectilinear	Limbless burrower	5,6,9	MS	2 (3D)	7.8-8.3
Lizard	*Bradypodion pumilum*	Chamaeleonidae	Intact limbs	Arboreal	Quadrupedal arboreal	Quadrupedal arboreal	5	MS	1 (3D)	12.7
Lizard	*Chalcides chalcides*	Scincidae	Strongly reduced limbs, elongated body	Facultative burrower	Slow lateral undulation	Limbless or limb-reduced facultative burrower	2-4,12,14	MS	2 (3D)	13.4-13.9
Lizard	*Chalcides sepsoides*	Scincidae	Strongly reduced forelimbs, elongated body	Facultative burrower	Slow lateral undulation	Limbless or limb-reduced facultative burrower	2-4,12,15	MS	1 (3D)	9.8
Lizard	*Trioceros jacksonii*	Chamaeleonidae	Intact limbs	Arboreal	Quadrupedal arboreal	Quadrupedal arboreal	16	FS	4 (3D;TR)	24.2-29.3
Lizard	*Dasia olivacea*	Scincidae	Intact limbs	Arboreal	Quadrupedal arboreal	Quadrupedal arboreal	17	FS	1 (3D)	22.8
Lizard	*Draco volans*	Agamidae	Intact limbs	Aerial/Arboreal	Quadrupedal arboreal with facultative aerial	Quadrupedal facultative bipedal/aerial	5,13	MS	2 (3D)	18.0-21.0
Lizard	*Eublepharis macularius*	Eublepharidae	Intact limbs	Terrestrial	Quadrupedal terrestrial	Quadrupedal terrestrial	5	FS	1 (3D)	17.6
Lizard	*Gekko gecko*	Gekkonidae	Intact limbs	Arboreal	Quadrupedal arboreal	Quadrupedal arboreal	18	FS	1 (3D)	29.7
Lizard	*Hemiergis quadrilineata*	Scincidae	Strongly reduced forelimbs, elongated body	Facultative burrower	Slow lateral undulation	Limbless or limb-reduced facultative burrower	2-4,19,20	FS	3 (3D;IHC)	5.5-6.9
Lizard	*Lepidothyris fernandi*	Scincidae	Intact limbs	Terrestrial	Quadrupedal terrestrial	Quadrupedal terrestrial	21	FS	4 (3D;IHC;TR)	23.0-24.1
Lizard	*Lygodactylus picturatus*	Gekkonidae	Intact limbs	Arboreal	Quadrupedal arboreal	Quadrupedal arboreal	22	FS	1 (3D)	9.5
Lizard	*Melanoseps loveridgei*	Scincidae	Limbless, elongated body, cylindrical skull	Burrower	Modified concertina or rectilinear	Limbless burrower	2-5,23	FS	3 (3D;IHC)	9.8-10.4
Lizard	*Ophiodes fragilis*	Anguidae	Limbless (vestige hindlimb), elongated body	Facultative burrower	Slow lateral undulation	Limbless or limb-reduced facultative burrower	2-4,16,24	MS	1 (3D)	15.9
Lizard	*Phelsuma grandis*	Gekkonidae	Intact limbs	Arboreal	Quadrupedal arboreal	Quadrupedal arboreal	5	FS	1 (3D)	20.2
Lizard	*Plestiodon marginatus*	Scincidae	Intact limbs	Terrestrial	Quadrupedal terrestrial	Quadrupedal terrestrial	25	MS, FS	3 (3D)	9.7-12.2
Lizard	*Pogona vitticeps*	Agamidae	Intact limbs	Terrestrial	Quadrupedal terrestrial	Quadrupedal terrestrial	5	FS	8 (3D;LS;IHC; TR)	19.9-22.8
Lizard	*Pseudopus apodus*	Anguidae	Limbless, elongated body	Terrestrial	Lateral undulation	Limbless multi-habitat lateral undulation	2-5,26	MS, FS	5 (3D;IHC;TR)	16.8-17.5
Lizard	*Rieppeleon brevicaudatus*	Chamaeleonidae	Intact limbs	Arboreal	Quadrupedal arboreal	Quadrupedal arboreal	27	FS	2 (3D)	12.0-15.4
Lizard	*Takydromus sexlineatus*	Lacertidae	Intact limbs	Semi-arboreal	Quadrupedal arboreal	Quadrupedal arboreal	5	FS	3 (3D)	12.9-13.6
Lizard	*Teratoscincus scincus*	Gekkonidae	Intact limbs	Terrestrial	Quadrupedal terrestrial	Quadrupedal terrestrial	28	MS	1 (3D)	12.8
Lizard	*Tropidurus torquatus*	Tropiduridae	Intact limbs	Semi-arboreal	Quadrupedal arboreal with facultative bipedal	Quadrupedal facultative bipedal/aerial	5,29	MS	1 (3D)	18.5
Snake	*Boaedon fuliginosus*	Lamprophiidae	Limbless	Terrestrial	Lateral undulation	Limbless multi-habitat lateral undulation	4,5,30,31	FS	6 (3D;LS;IHC)	11.9-12.9
Snake	*Cerastes cerastes*	Viperidae	Limbless	Terrestrial	Sidewinding	Limbless multi-habitat other movements	30-32	MS	1 (3D)	12.2
Snake	*Chrysopelea ornata*	Colubridae	Limbless	Aerial/Arboreal	Lateral undulation	Limbless multi-habitat lateral undulation	13,33	MS, FS	4 (3D;IHC;TR)	21.0-21.4
Snake	*Dasypeltis gansi*	Colubridae	Limbless	Arboreal	Arboreal concertina	Limbless multi-habitat other movements	4,5,30,31	FS	4 (3D;IHC;TR)	9.4-9.8
Snake	*Dendrelaphis pictus*	Colubridae	Limbless	Arboreal	Arboreal concertina	Limbless multi-habitat other movements	4,5,30,31	MS	2 (3D)	13.6-15.5
Snake	*Epicrates cenchria*	Boidae	Limbless (vestigial hindlimb)	Terrestrial	Rectilinear	Limbless multi-habitat other movements	34,35	FS	3 (3D;IHC)	25.8-28.0
Snake	*Eryx colubrinus*	Boidae	Limbless (vestigial hindlimb)	Facultative Burrower	Slow lateral undulation	Limbless or limb-reduced facultative burrower	36,37	FS	3 (IHC;TR)	10.6-11.8
Snake	*Eryx jaculus*	Boidae	Limbless (vestigial hindlimb)	Facultative Burrower	Slow lateral undulation	Limbless or limb-reduced facultative burrower	36,37	MS	3 (3D)	9.5-12.2
Snake	*Hydrophis platurus*	Elapidae	Limbless	Aquatic	Lateral undulation	Limbless multi-habitat lateral undulation	4,5,38	MS	3 (3D)	17.0-17.9
Snake	*Pantherophis guttatus*	Colubridae	Limbless	Terrestrial	Lateral undulation	Limbless multi-habitat lateral undulation	34,39	FS	4 (3D;TR)	13.5-18.0
Snake	*Python regius*	Pythonidae	Limbless (vestigial hindlimb)	Terrestrial	Rectilinear	Limbless multi-habitat other movements	22,30,31,3 5	FS	3 (3D;IHC;TR)	25.2-26.0
Snake	*Xerotyphlops vermicularis*	Typhlopidae	Limbless (vestigial hindlimb), cylindrical skull	Burrower	Modified concertina or rectilinear	Limbless burrower	5,6,40,41	MS, FS	4 (3D)	5.6-6.3

*Source of specimens: MS=Museum specimen; FS=Freshly dissected sample
†Total number of specimens analyzed and their experimental destination: 3D=3D models; LS=light-sheet microscopy; IHC=immunohistochemistry; TR=transcriptome
‡Size range (snout to atlanto-occipital junction length, mm) of individuals analyzed

Supplementary Table 2: Estimation of phylogenetic signal using a multivariate *K*-statistic

Partition	K_{mult}	*p*-value*
Whole-brain	0.6903	< 0.001
Cerebellum (20 landmarks)	0.5753	< 0.001
Cerebellum (15 landmarks)	0.5684	< 0.001
Telencephalon	0.7118	< 0.001
Diencephalon	0.5653	< 0.001
Mesencephalon	0.5486	< 0.001
Medulla oblongata	0.6188	< 0.001

*p-values from permutation tests (10,000 permutation rounds).

Supplementary Table 3: Phylogenetic ANOVA for the whole brain and each brain subdivision

Partition	Effect	df[*]	SS[†]	MS[‡]	Rsq	F	Z	*p*-value[§]
Whole-brain	Locomotion	6	0.0020	0.0003	0.1724	1.1455	-0.8931	0.8076
	Residuals	33	0.0095	0.0003				
	Total	39	0.0115					
Cerebellum (20 landmarks)	Locomotion	6	0.0069	0.0011	0.3411	2.8473	2.4326	**0.0046**
	Residuals	33	0.0133	0.0004				
	Total	39	0.0202					
Cerebellum (15 landmarks)	Locomotion	6	0.0064	0.0011	0.3111	2.4835	1.8066	**0.0333**
	Residuals	33	0.0141	0.0004				
	Total	39	0.0205					
Telencephalon	Locomotion	6	0.0018	0.0003	0.1645	1.0830	-0.8382	0.7932
	Residuals	33	0.0093	0.0003				
	Total	39	0.0111					
Diencephalon	Locomotion	6	0.0027	0.0004	0.1692	1.1197	-0.7952	0.7861
	Residuals	33	0.0132	0.0004				
	Total	39	0.0158					
Mesencephalon	Locomotion	6	0.0029	0.0005	0.1828	1.2306	-0.6429	0.7356
	Residuals	33	0.0128	0.0004				
	Total	39	0.0156					
Medulla oblongata	Locomotion	6	0.0012	0.0002	0.1759	1.1741	-0.6857	0.7524
	Residuals	33	0.0055	0.0002				
	Total	39	0.0067					

[*]df, degree of freedom.
[†]SS, sum of squares.
[‡]MS, mean squares.
[§]Significant values (*p*-values < 0.05) from permutation tests (10,000 permutation rounds) are bolded.

Supplementary Table 4: Pairwise differences from phylogenetic ANOVA for the cerebellum

Cerebellum (20 landmarks)

Locomotor modes*, †	Lbu‡	Llu‡	Lo‡	Lfbu‡	Qte‡	Qar‡	Qf‡
Lbu‡	-	**2.7962**	**3.0696**	**2.3137**	**3.4003**	**4.0491**	**3.4467**
Llu‡	0.0075	-	-1.0082	**1.6509**	**3.1285**	**4.3841**	**2.5178**
Lo‡	0.0039	0.8441	-	**2.5676**	**4.0765**	**5.1116**	**3.2212**
Lfbu‡	0.0216	0.0627	0.0132	-	**1.5889**	**3.3251**	1.5789
Qte‡	0.0016	0.0048	0.0005	0.0482	-	-0.2062	-0.4417
Qar‡	0.0004	0.0002	0.0001	0.0023	0.5385	-	-0.6233
Qf‡	0.0016	0.0147	0.0023	0.0707	0.6429	0.7119	-

Cerebellum (15 landmarks)

Locomotor modes*, †	Lbu‡	Llu‡	Lo‡	Lfbu‡	Qte‡	Qar‡	Qf‡
Lbu‡	-	**3.4763**	**4.0718**	**2.6734**	**1.1893**	**4.1564**	**3.4188**
Llu‡	0.0017	-	-1.0066	**1.8128**	**2.9312**	**3.8768**	**2.2307**
Lo‡	0.0001	0.8444	-	**2.9502**	**4.0798**	**4.6832**	**3.0239**
Lfbu‡	0.0116	0.0470	0.0060	-	1.4608	**3.1970**	1.4151
Qte‡	0.0037	0.0069	0.0004	0.0831	-	0.7254	0.2213
Qar‡	0.0005	0.0004	0.0003	0.0035	0.2253	-	0.6463
Qf‡	0.0022	0.0259	0.0046	0.0898	0.5527	0.7227	-

*Effect sizes (Z-scores) and associated *p*-values are indicated in upper and lower off-diagonal cells, respectively.
†Significant Z-score values (*p*-values < 0.05) from permutation tests (10,000 permutation rounds) are bolded.
‡Locomotor modes: limbless or limb-reduced burrower (Lbu); limbless or limb-reduced multi-habitat lateral undulation (Llu); limbless or limb-reduced multi-habitat other movements (Lo); limbless or limb-reduced facultative burrower (Lfbu); quadrupedal terrestrial (Qte); quadrupedal arboreal (Qar); quadrupedal facultative bipedal/aerial (Qf).

Supplementary Table 5: Convergence metrics for the cerebellum in groups with lizard and snake species

Locomotor modes	C1*	*p*-value	C2*	*p*-value	C3*	*p*-value	C4*	*p*-value
Lbu†	**0.1302**	0.0100	**0.0425**	0.0020	**0.0636**	0.0120	**0.0062**	0.0090
Lfbu†	**0.1328**	0.0050	**0.0310**	0.0110	**0.0557**	0.0130	**0.0046**	0.0360
Llu†	0.0106	0.8671	0.0016	0.0905	0.0046	0.8911	0.0002	0.9271

*Significant values (*p*-values < 0.05) from 1,000 evolutionary simulations are bolded.
†Locomotor modes: limbless or limb-reduced burrower (Lbu); limbless or limb-reduced facultative burrower (Lfbu); limbless or limb-reduced multi-habitat lateral undulation (Llu).

Supplementary Table 6: Pairwise comparisons of regression slopes

Pairwise comparisons	Difference in slopes	95% LCI†	95% UCI‡	*p*-unadjusted	*p*-adjusted §
Llu *vs* Lbu*	-1.21236	-2.10731	-0.31741	0.00986	0.16762
Lo *vs* Lbu*	-1.26428	-1.83239	-0.69618	0.00010	0.00200
Lfbu *vs* Lbu*	-1.42205	-1.96521	-0.87889	0.00001	0.00021
Qf *vs* Lbu*	-1.16252	-2.27650	-0.04853	0.04146	0.66336
Qar *vs* Lbu*	-1.07856	-1.61019	-0.54694	0.00030	0.00570
Qte *vs* Lbu*	-1.01544	-1.55070	-0.48019	0.00061	0.01098
Lo *vs* Llu*	-0.05192	-0.91460	0.81076	0.90249	1.00000
Lfbu *vs* Llu*	-0.20969	-1.05615	0.63677	0.61490	1.00000
Qf *vs* Llu*	0.04984	-1.23951	1.33919	0.93727	1.00000
Qar *vs* Llu*	0.13379	-0.70531	0.97290	0.74572	1.00000
Qte *vs* Llu*	0.19691	-0.64450	1.03833	0.63450	1.00000
Lfbu *vs* Lo*	-0.15777	-0.64593	0.33039	0.51233	1.00000
Qf *vs* Lo*	0.10177	-0.98646	1.18999	0.84906	1.00000
Qar *vs* Lo*	0.18572	-0.28958	0.66102	0.42916	1.00000
Qte *vs* Lo*	0.24884	-0.23052	0.72819	0.29576	1.00000
Qf *vs* Lfbu*	0.25954	-0.81588	1.33495	0.62401	1.00000
Qar *vs* Lfbu*	0.34349	-0.10170	0.78867	0.12484	1.00000
Qte *vs* Lfbu*	0.40661	-0.04291	0.85612	0.07433	1.00000
Qar *vs* Qf*	0.08395	-0.98569	1.15359	0.87308	1.00000
Qte *vs* Qf*	0.14707	-0.92437	1.21851	0.78006	1.00000
Qte *vs* Qar*	0.06312	-0.37239	0.49863	0.76814	1.00000

* Locomotor modes: limbless or limb-reduced burrower (Lbu); limbless or limb-reduced multi-habitat lateral undulation (Llu); limbless or limb-reduced multi-habitat other movements (Lo); limbless or limb-reduced facultative burrower (Lfbu); quadrupedal terrestrial (Qte); quadrupedal arboreal (Qar); quadrupedal facultative bipedal/aerial (Qf).

†95% Lower confidence interval value

‡95% Upper confidence interval value

§Significant *p*-values corrected with the Holm-Bonferroni method

Supplementary Table 7: Pairwise differences from Johnson-Neyman procedure

Significant pairwise comparisons	Inferior limit whole-brain (mm3) †	Superior limit whole-brain (mm3) †
Lbu *vs* Llu*	1.9485	5.5906
Lbu *vs* Lo*	1.4025	6.7946
Lbu *vs* Lfbu*	1.4933	6.8042
Lbu *vs* Qf*	0.7187	8.2505
Lbu *vs* Qar*	2.4225	6.8277
Lbu *vs* Qte*	1.9117	7.4338
Llu *vs* Lo*	0	6.7546
Llu *vs* Lfbu*	0	7.6275
Llu *vs* Qf*	0	5.1481
Llu *vs* Qar*	0	4.0219
Llu *vs* Qte*	0	4.0140
Lo *vs* Lfbu*	0	12.2831
Lo *vs* Qf*	0	8.4762
Lo *vs* Qar*	0	7.8398
Lo *vs* Qte*	0	6.8677
Lfbu *vs* Qf*	0	9.6744
Lfbu *vs* Qar*	0	7.7932
Lfbu *vs* Qte*	0	7.1022
Qf *vs* Qar*	0	11.8409
Qf *vs* Qte*	0	11.0250
Qar *vs* Qte*	0	11.9008

*Locomotor modes: limbless or limb-reduced burrower (Lbu); limbless or limb-reduced multi-habitat lateral undulation (Llu); limbless or limb-reduced multi-habitat other movements (Lo); limbless or limb-reduced facultative burrower (Lfbu); quadrupedal terrestrial (Qte); quadrupedal arboreal (Qar); quadrupedal facultative bipedal/aerial (Qf).

†Regions of non-significance are indicated based on plot in Fig. 3a.

Supplementary Table 8: One-way ANOVAs showing 3D-digitizing errors relative to individual differences

Data	Effect	SS*	MS†	df‡	F	*p*-value
Shape whole-brain	Individual	3.0188	0.0004	6864	1576.21	<0.0001
Shape cerebellum (20 landmarks)	Digitizing error	4.9885	0.0024	2067	1550.60	<0.0001
Centroid size whole-brain	Individual	4819022511.22	123564679.77	39	965295.92	<0.0001
Centroid size cerebellum (20 landmarks)	Digitizing error	316898949.39	8125614.09	39	457550.17	<0.0001

*SS, sum of squares.
†MS, mean squares.
‡df, degree of freedom.

Supplementary References

1. Böhme, W. et al. *Ablepharus kitaibelii* (amended version of 2009 assessment). *The IUCN Red List of Threatened Species* **2017**, e.T29691A115767606 (2017).
2. Gans, C. *Biomechanics: approach to vertebrate biology*. Lippincott, Philadelphia (1974).
3. Gans, C. Tetrapod limblessness: evolution and functional corollaries. *Amer. Zool.* **15**, 455-467 (1975).
4. Gans, C. Locomotion of limbless vertebrates: pattern and evolution. *Herpetologica* **42**, 33-46 (1986).
5. Da Silva, F. O. et al. The ecological origins of snakes as revealed by skull evolution. *Nat. Commun.* **9**, 376 (2018).
6. Gans, C. Approaches to the evolution of limbless locomotion. *Cuad. Herpetol.* **8**, 12-17 (1994).
7. Leonard, C. J. Locomotion of the limbless skink *Acontias meleagris*. *J. Herp. Assoc. Africa* **36**, 73-73 (1989).
8. Kearney, M. Systematics of the Amphisbaenia (Lepidosauria: Squamata) based on morphological evidence from recent and fossil forms. *Herpet. Monog.* **17**, 1-74 (2003).
9. Jayne B. C. Kinematics of terrestrial snake locomotion. *Copeia* **4**, 915-927 (1986).
10. Gasc, J. P. & Gans, C. Tests on locomotion of the elongate and limbless lizard *Anguis fragilis* (Squamata: Anguidae). *Copeia* **4**, 1055-1067 (1990).
11. Avila-Pires, T. C. S. *Bachia flavescens*. *The IUCN Red List of Threatened Species* **2010**, e.T178655A7589472 (2010).
12. Greer, A. E., Caputo, V., Lanza, B. & Palmieri, R. Observations on limb reduction in the scincid lizard genus *Chalcides*. *J. Herpetol.* **32**, 244-252 (1998).
13. R. McNeill, A. Locomotion of reptiles. *Herpetol. Bull.* **121**, 1-5 (2012).
14. Rugiero, L. On the ecology and phenology of *Chalcides chalcides* (LINNAEUS, 1758) in Central Italy. *Herpetozoa* **10**, 81-84 (1997).
15. Disi, M., A. M., Werner, Y. & El Din, S. B. *Chalcides sepsoides*. *The IUCN Red List of Threatened Species* **2006**, e.T61571A12518031 (2006).
16. Pianka, E. R. & Vitt, P. L. *Lizards: windows to the evolution of diversity*. University of California Press (2003).
17. Inger, R. F. & Stuart, B. L. *Dasia olivacea*. *The IUCN Red List of Threatened Species* **2010**, e.T178252A7507427 (2010).
18. Heinkel, F. W. & Schmidt, W. *Geckoes*. Krieger Publishing Co. Malabar, FL (1995).
19. Davis, R. A. & Doherty, T. S. Rapid recovery of an urban remnant reptile community following summer wildfire. *PLoS One* **10**, e0127925 (2015).
20. Shapiro, M. S. Developmental morphology of limb reduction in *Hemiergis* (Squamata: Scincidae): chondrogenesis, osteogenesis, and heterochrony. *J. Morphol.* **254**, 211-31 (2002).
21. Akani, G. C., Capizzi, D. & Luiselli, L. Community ecology of scincid lizards in a swamp rainforest of South-Eastern Nigeria. *Russ. J. Herpetol.* **9**, 125-134 (2002).
22. Spawls, S., Howell, K., Drewes, R. C. & Ashe, J. *A field guide to the reptiles of East Africa*. Academic Press (2002).
23. Malonza, P. K. & Bwong, B. A. Life history notes on loveridge's limbless skink *Melanoseps loveridgei* Brygoo & Roux-Esteve, 1981 (Sauria: Scincidae: Feylininae). *Herpetotropicos* **5**, 115-117 (2011).
24. de Barros, E. H. & Teixeira, R. L. Diet and fecundity of the glass-lizard, *Ophiodes striatus* (Sauria, Anguidae) from the Atlantic Forest in southeastern Brazil. *Bol. Mus. Biol. Mello Leitão* **22**, 11-23(2007).
25. Kidera, N. & Ota, H. *Plestiodon marginatus*. *The IUCN Red List of Threatened Species* **2017**, e.T96265367A96265370 (2017).
26. Ganc, C. & Gasc, J.P. Tests of locomotion of the elongate and limbless reptile *Ophisaurus apodus* (Sauria: Anguidae). *J. Zool. Lond.* **220**, 517-536 (1990).
27. Tolley, K. & Menegon, M. *Rieppeleon brevicaudatus*. *The IUCN Red List of Threatened Species* **2014**, e.T172520A1344134 (2014).
28. Carranza, S. et al. Diversity, distribution and conservation of the terrestrial reptiles of Oman (Sauropsida, Squamata). *PLoS One* **13**, e0190389 (2018).
29. Rocha-Barbosa, O., Loguercio, M. F., Velloso, A.L. & Bonates, A. C. Bipedal locomotion in Tropidurus torquatus (Wied, 1820) and Liolaemus lutzae Mertens, 1938. *Braz. J. Biol.* **68**, 649-55 (2008).

30. Gray, J. The mechanism of locomotion in snakes. *J. Exp. Biol.* **23**, 101-120 (1946).
31. Gray, J. *Animal locomotion*. Weidenfeld and Nicolson, London (1968).
32. Phelps, T. *Old world vipers: a natural history of the Azemiopinae and Viperinae*. Edition Chimaira, Frankfurt and Main, Germany (2010).
33. Whitaker, R., Captain, A. & Ahmed, F. *Snakes of India*. Draco Books (2004).
34. Lawing, A. M., Head, J. J. & Polly, P. D. The ecology of morphology: the ecometrics of locomotion and macroenvironment in North American snakes. In: Louys, L. (ed). *Paleontology in Ecology and Conservation*. Springer-Verlag, Berlin and Heidelberg (2012).
35. Bogert, C. Rectilinear locomotion in snakes. *Copeia* **4**, 253-254 (1947).
36. Kreiner, G. *The snakes of Europe: all species from west of the Caucasus mountains*. Edition Chimaria, Frankfurt am Main (2007).
37. Zheng, L. et al. Coupling effect of morphology and mechanical properties contributes to the tribological behaviors of snake scales. *J. Bionic Eng.* **15**, 481-493 (2018).
38. Brischoux, F. & Shine, R. Morphological adaptations to marine life in snakes. *J. Morphol.* **272**, 566-572 (2011).
39. Jayne, B. C. Swimming in constricting (*Elaphe g. guttata*) and nonconstricting (*Nerodia fasciata pictiventris*) colubrid snakes. *Copeia* **1**, 195-208 (1985).
40. Hedges, S. B., Marion, A. B., Lipp, K. M., Marin, J. & Vidal, N. A taxonomic framework for typhlopid snakes from the Caribbean and other regions (Reptilia, Squamata). *Caribb. Herpetol.* **49**, 1-61 (2014).
41. Ehmann, H. & Bamford, M. J. Family Typhlopidae. In: Glasby, C.J., Ross, G.J.B. & Beesley, P.L. (eds). *Fauna of Australia 2A*. AGPS Canberra (1993).
