# Supplementary material for: Comparative analysis of squamate brains unveils multi-level variation in cerebellar architecture associated with locomotor specialization
Source: Nat Commun. 2019 Dec 5;10:5560. doi: 10.1038/s41467-019-13405-w (PMC6895188; doi:10.1038/s41467-019-13405-w)
Supplement: Supplementary file 3 — Reporting Summary [file 41467_2019_13405_MOESM3_ESM.pdf]

nature research

Corresponding author(s): Di-Poï Nicolas

Last updated by author(s): Oct 30, 2019

Reporting Summary

Nature Research wishes to improve the reproducibility of the work that we publish. This form provides structure for consistency and transparency in reporting. For further information on Nature Research policies, see Authors & Referees and the Editorial Policy Checklist.

Statistics

For all statistical analyses, confirm that the following items are present in the figure legend, table legend, main text, or Methods section.

n/a	Confirmed	
☐	☒	The exact sample size (*n*) for each experimental group/condition, given as a discrete number and unit of measurement
☐	☒	A statement on whether measurements were taken from distinct samples or whether the same sample was measured repeatedly
☐	☒	The statistical test(s) used AND whether they are one- or two-sided<br>*Only common tests should be described solely by name; describe more complex techniques in the Methods section.*
☐	☒	A description of all covariates tested
☐	☒	A description of any assumptions or corrections, such as tests of normality and adjustment for multiple comparisons
☐	☒	A full description of the statistical parameters including central tendency (e.g. means) or other basic estimates (e.g. regression coefficient) AND variation (e.g. standard deviation) or associated estimates of uncertainty (e.g. confidence intervals)
☐	☒	For null hypothesis testing, the test statistic (e.g. *F*, *t*, *r*) with confidence intervals, effect sizes, degrees of freedom and *P* value noted<br>*Give P values as exact values whenever suitable.*
☒	☐	For Bayesian analysis, information on the choice of priors and Markov chain Monte Carlo settings
☐	☒	For hierarchical and complex designs, identification of the appropriate level for tests and full reporting of outcomes
☐	☒	Estimates of effect sizes (e.g. Cohen's *d*, Pearson's *r*), indicating how they were calculated

Our web collection on statistics for biologists contains articles on many of the points above.

Software and code

Policy information about availability of computer code

Data collection	No unreported software was used

Data analysis

All packages and softwares used have been already described and are mentioned in the Methods section of the manuscript:
-R-package pca3d v0.10 (https://CRAN.R-project.org/package=pca3d)
-R-package geomorph v3.0.7 (https://CRAN.R-project.org/package=geomorph)
-R-package convevol v1.3 (https://CRAN.R-project.org/package=convevol)
-R-package Morpho v2.6 (https://CRAN.R-project.org/package=Morpho)
-R-package Rvcg v0.18 (https://CRAN.R-project.org/package=Rvcg)
-R-package viridis v0.5.1 (https://CRAN.R-project.org/package=viridis)
-R-package phylolm v2.6 (https://CRAN.R-project.org/package=phylolm)
-Amira 5.5.0 software (Thermo Fisher Scientific)
-GPSA software (http://morphlab.sc.fsu.edu/software/gpsa/index.html)
-ZEN software (Zeiss, Germany)
-Imaris Image Stitcher application (Bitplane, Switzerland)
-Imaris v9.2 software (Bitplane, Switzerland)
-Fiji package (https://imagej.net/Fiji)
-R-package ggplot2 v3.1.0 (https://CRAN.R-project.org/package=ggplot2)
-Trimmomatic tool (http://www.usadellab.org/cms/?page=trimmomatic)
-Trinity software v2.8.5 (https://github.com/trinityrnaseq/trinityrnaseq/releases)
-Blast2GO program (BioBam)
-OrthoFinder algorithm (https://github.com/davidemms/OrthoFinder)
-Expectation Maximization (RSEM) software (https://deweylab.github.io/RSEM/)
-Heatmapper software (http://www.heatmapper.ca/)
-R-package pvclust v2.0-0 (https://CRAN.R-project.org/package=pvclust)
-R-package GOstats (https://www.bioconductor.org/packages/release/bioc/html/GOstats.html)

For manuscripts utilizing custom algorithms or software that are central to the research but not yet described in published literature, software must be made available to editors/reviewers. We strongly encourage code deposition in a community repository (e.g. GitHub). See the Nature Research guidelines for submitting code & software for further information.

Data

Policy information about availability of data

All manuscripts must include a data availability statement. This statement should provide the following information, where applicable:

- Accession codes, unique identifiers, or web links for publicly available datasets
- A list of figures that have associated raw data
- A description of any restrictions on data availability

Landmarks and main PC scores used for visualization and analysis are available in the main Figures and Supplementary Information. Illumina reads and processed RNA sequencing data have been deposited on the Gene Expression Omnibus (GEO) database under the accession number GSE139570 [https://www.ncbi.nlm.nih.gov/geo/query/acc.cgi?acc=GSE139570]. Source data on geometric morphometrics for Figures 3-5, Purkinje cell counts for Figure 7c, and gene expression for Figure 8a are provided as a Source Data file. 3D brain models are available through the corresponding author, upon reasonable request.

Field-specific reporting

Please select the one below that is the best fit for your research. If you are not sure, read the appropriate sections before making your selection.

☒ Life sciences ☐ Behavioural & social sciences ☐ Ecological, evolutionary & environmental sciences

For a reference copy of the document with all sections, see nature.com/documents/nr-reporting-summary-flat.pdf

Life sciences study design

All studies must disclose on these points even when the disclosure is negative.

Sample size	Our study covers all major groups of lizards and snakes. The number of non-model organisms analyzed was limited by sampling difficulties for some particular lizard and snake species, including species with particular ecological behaviours and/or a geographically restricted area of distribution. An average of 2.6 individuals per species was examined in our study (range 1-8 depending on sample availability and number of analyses performed), and specimens were used for multiple observations and/or analyses whenever possible.
Data exclusions	No data excluded.
Replication	Different methods, species datasets, and/or landmarks were used to ensure the validity and reproducibility of our results.
Randomization	Species were grouped according to their main ecology (locomotory behaviour), because the main goal of this study was to compare groups of individuals belonging to different species with specific locomotor behaviours and covering the entire squamate phylogeny .
Blinding	Species were initially selected to cover all major groups of squamates, and then grouped according to their main ecology (locomotory behaviour) before running any analysis, so no blinding was necessary during data analysis.

Reporting for specific materials, systems and methods

We require information from authors about some types of materials, experimental systems and methods used in many studies. Here, indicate whether each material, system or method listed is relevant to your study. If you are not sure if a list item applies to your research, read the appropriate section before selecting a response.

Materials & experimental systems

n/a	Involved in the study
☐	☒ Antibodies
☒	☐ Eukaryotic cell lines
☒	☐ Palaeontology
☐	☒ Animals and other organisms
☒	☐ Human research participants
☒	☐ Clinical data

Methods

n/a	Involved in the study
☒	☐ ChIP-seq
☒	☐ Flow cytometry
☒	☐ MRI-based neuroimaging

Antibodies

Antibodies used	-Calbindin D-28K primary antibody (dilution 1:300, rabbit polyclonal, Swant, Switzerland, cat# CB38, RRID: AB_10000340)<br>-Zinc finger proteins 1/2/3 primary antibody (dilution 1:300, rabbit polyclonal, LifeSpan BioSciences, U.S.A., cat# LS-C118695)<br>-Alexa Fluor-conjugated secondary antibody (dilution 1:500, goat anti-rabbit IgG, Thermo Fisher Scientific, U.S.A., cat# A-11008, RRID: AB_143165)
Validation	Only antibodies validated in previous publications and exhibiting a broad cross-reactivity (including in chicken and/or reptiles) were used. Negative controls were also performed to ensure specificity of primary antibodies in immunohistochemistry experiments.

Animals and other organisms

Policy information about studies involving animals; ARRIVE guidelines recommended for reporting animal research

Laboratory animals	All squamate species (29 lizards and 11 snakes) were obtained at young adult stage from collections at the Finnish Museum of Natural History (Finland), reptile colonies at the University of Helsinki (Finland), collaborators (Michael D. Shapiro, University of Utah, U.S.A.), and specialized retailers. All specimens are listed in Supplementary Table 1.
Wild animals	Only gravid Hemiergis quadrilineata specimens were captured in the wild (see licence below), collected animals were not killed.
Field-collected samples	Hemiergis quadrilineata specimens were collected under Licences to Take Fauna for Scientific Purposes from the Western Australian Department of Conservation and Land Management (No. SF003009 to Michael D. Shapiro) and exported under Environment Australia (EA) Permit to Export (No. PWS 994167; EA Approved Institution Number AI1146).
Ethics oversight	All reptile captive breedings and experiments were approved by the Laboratory Animal Centre (LAC) of the University of Helsinki and/or the National Animal Experiment Board (ELLA) in Finland (license numbers ESLH-2007-07445/ym-23, ESAVI/7484/04.10.07/2016, and ESAVI/13139/04.10.05/2017).

Note that full information on the approval of the study protocol must also be provided in the manuscript.
